# Distinct Subsets of Lateral Hypothalamic Neurotensin Neurons are Activated by Leptin or Dehydration

**DOI:** 10.1038/s41598-018-38143-9

**Published:** 2019-02-12

**Authors:** Juliette A. Brown, Anna Wright, Raluca Bugescu, Lyndsay Christensen, David P. Olson, Gina M. Leinninger

**Affiliations:** 1 Michigan State University Department of Pharmacology and Toxicology, East Lansing, 48824 MI USA; 2 Institute for Integrative Toxicology at Michigan State University, East Lansing, 48824 MI USA; 3 Michigan State University Department of Physiology, East Lansing, 48824 MI USA; 4 Division of Metabolism, Endocrinology and Diabetes, Department of Internal Medicine, University of Michigan, Ann Arbor, MI 48109 USA; 5 Department of Pediatrics and Communicable Diseases, University of Michigan, Ann Arbor, MI 48109 USA

## Abstract

The lateral hypothalamic area (LHA) is essential for ingestive behavior but it remains unclear how LHA neurons coordinate feeding vs. drinking. Most LHA populations promote food and water consumption but LHA neurotensin (Nts) neurons preferentially induce water intake while suppressing feeding. We identified two molecularly and projection-specified subpopulations of LHA Nts neurons that are positioned to coordinate either feeding or drinking. One subpopulation co-expresses the long form of the leptin receptor (LepRb) and is activated by the anorectic hormone leptin (Nts^LepRb^ neurons). A separate subpopulation lacks LepRb and is activated by dehydration (Nts^Dehy^ neurons). These molecularly distinct LHA Nts subpopulations also differ in connectivity: Nts^LepRb^ neurons project to the ventral tegmental area and substantia nigra compacta but Nts^Dehy^ neurons do not. Intriguingly, the LHA Nts subpopulations cannot be discriminated via their classical neurotransmitter content, as we found that all LHA Nts neurons are GABAergic. Collectively, our data identify two molecularly- and projection-specified subpopulations of LHA Nts neurons that intercept either leptin or dehydration cues, and which conceivably could regulate feeding vs. drinking behavior. Selective regulation of these LHA Nts subpopulations might be useful to specialize treatment for ingestive disorders such as polydipsia or obesity.

## Introduction

The lateral hypothalamic area (LHA) of the brain receives inputs from osmotic and energy-sensing sites and projects to centers coordinating goal-directed ingestive behavior to maintain homeostasis^1–6^. Early studies defined the LHA as a “feeding center” because animals with lesion of LHA cell bodies lost all motivation to eat^7,8^. Less emphasized, but equally important, is that animals with LHA lesions also lost the motivation to drink water and their resulting dehydration causes death well before starvation^9,10^. Intriguingly, destroying passing dopaminergic fibers within the LHA similarly produces aphagia and adipsia, revealing that the LHA acts in concert with the dopamine system^11^. Thus, the LHA modifies both types of ingestive behavior necessary for survival, but via incompletely understood mechanisms.

The discovery of molecularly- and projection-specified populations of neurons within the LHA suggested that some of them might be specialized to coordinate drinking vs. feeding. Yet, most of the LHA populations studied to date indiscriminately promote intake of food and water. LHA neurons expressing melanin concentrating hormone promote intake of both substances and do not specifically organize feeding vs. drinking^12,13^. A separate population of orexin/hypocretin-expressing LHA neurons regulate arousal-dependent behaviors, including feeding, drinking and locomotor activity, but do not specify a particular ingestive behavior^14–16^. LHA neurons have also been distinguished by their expression of the classical neurotransmitters glutamate or GABA. Inhibiting LHA glutamate neurons increases intake of a palatable “meal replacement” drink^17^, but it is unclear if this is an effort to obtain fluid, calories or if both ingestive behaviors are modulated by these neurons. Activation of all LHA GABA neurons increases behaviors to obtain food and liquids, but also invokes gnawing at non-caloric objects such as wood or the cage floor^18–20^; thus, bulk activation of LHA GABA neurons cannot be considered to direct any specific ingestive behavior. While en masse activation of LHA GABA neurons is unlikely to occur in nature, there are subpopulations of LHA GABAergic neurons^18^ that may be activated by different physiologic cues to control intake. For example, LHA GABA neurons co-expressing the neuropeptide galanin mediate food seeking^21^. Conversely, the subset of LHA GABA neurons co-expressing the long form of the leptin receptor (LepRb) limit feeding with no effect on water intake^22,23^. It is therefore possible that subsets of LHA GABA neurons might be activated by distinct physiologic cues, and hence differentially control food vs. water intake. However, the studies of LHA populations to date do not explain how the LHA specifically coordinates feeding or osmolality cues to direct the appropriate ingestive behavior.

We recently characterized a large population of LHA neurons that express the neuropeptide Neurotensin (Nts) and are separate from MCH or orexin/hypocretin neurons^22,24^. Unlike other LHA populations that promote both food and water consumption, experimental activation of LHA Nts neurons promotes voracious drinking but restrains feeding^24^. Since LHA Nts neurons have been reported to contain glutamate^25^ or GABA^26,27^, we hypothesized that there might be neurochemically, molecularly, and functionally heterogeneous subsets of LHA Nts neurons to coordinate drinking vs. feeding. Indeed, some (but not all) LHA Nts neurons co-express the long form of the leptin receptor (LepRb) and GABA and are activated by the anorectic hormone leptin^21,26^; we refer to these as Nts^LepRb^ neurons. This Nts^LepRb^ population comprises a small, but essential subset of LHA Nts neurons necessary to mediate the anorectic response to leptin and proper regulation of energy balance^22^. Yet, mice lacking LepRb in LHA Nts^LepRb^ neurons do not exhibit any disruptions in drinking or bodily fluid content, suggesting that LHA Nts-mediated drinking might be mediated via different LHA Nts neurons^22^. Some LHA Nts neurons are responsive to physiologic changes in serum osmolality, as dehydration increases expression of Nts mRNA within the LHA^28^; we refer to these as Nts^Dehy^ neurons. Exogenous Nts treatment also promotes drinking^29^, although the endogenous sources of Nts mediating this effect remained unknown. Given that experimental activation of LHA Nts neurons promotes Nts release^24,27^ and drinking^24,27,30,31^, the dehydration-induced upregulation of LHA Nts could serve as a physiologic signal to drive water seeking and intake once water becomes available^32^. Taken together, these data suggest that some LHA Nts neurons can be activated by cues of energy or osmolality status and might comprise separate populations to coordinate feeding or drinking behavior. We therefore assessed whether LHA Nts^LepRb^ neurons and Nts^Dehy^ neurons are the same, or whether they are separate populations that are distinguishable via molecular, circuit and neurotransmitter criteria.

## Results

### Methods to Identify LHA Nts Neurons

**Figure 1:**
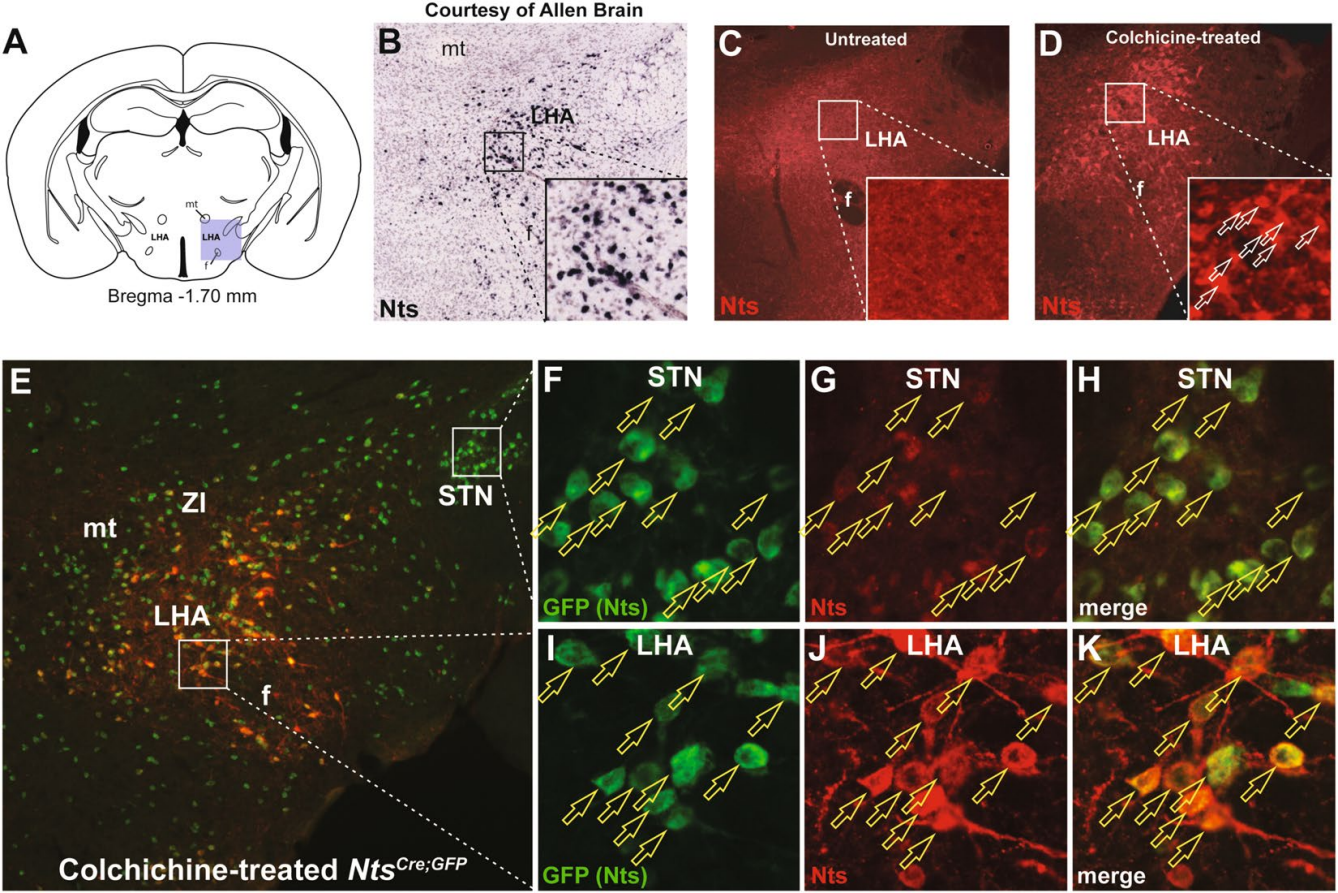
Visualization of LHA Nts Neurons. (**A**) Coronal schematic from mouse brain atlas. The shaded square identifies the portion of the LHA shown in subsequent microscopy panels. (**B**) *Nts*-expressing cell bodies detected via ISH (courtesy of the Allen Brain Atlas^36^). (**C**) Nts-Immunofluoresence (IF) only identifies fibers within the LHA unless (**D**) mice were pretreated with ICV colchicine, which inhibits axonal transport and permits detection of Nts-IF within cell bodies (white outline arrows). (**E**) Nts-IF (red) in colchicine-treated *Nts*^*Cre*^; *GFP* mouse that expresses GFP in Nts neurons (green). (**F**–**H**) Insets show the STN from E. (**F**) Many GFP-labeled Nts cell bodies are found within the STN consistent with *Nts*-ISH^36^ and (**G**) the Nts-IF cell bodies in this region (**H**) entirely overlap with the GFP (Nts) cells (yellow outline arrows). (**I**–**K**) Insets show the LHA from (**E**), where (**I**) the GFP-labeled cell bodies and (**J**) Nts-IF cell bodies (**K**) overlap (yellow outline arrows). Together these data confirm that *Nts*^*Cre*^; *GFP* mice correctly identify Nts-expressing cells, and can be used to visualize them.

*In situ* hybridization (ISH) identifies many Nts-expressing cells within the LHA (Fig. 1A,B)^28^ but does not easily permit determination of co-expressed transcripts or circuit tracing. Nts immunofluorescence (Nts-IF) permits such analyses, but only labels fibers, not cell bodies (Fig. 1C) unless mice were pre-treated with the axonal transport inhibitor, colchicine (Fig. 1D)^33,34^. Since colchicine induces neuronal dysfunction and lethality its use prohibits study of Nts contributions to normal physiology. Nonetheless, Nts-ISH and -IF confirm a large population of LHA neurons that actively express Nts (Fig. 1B,D). We reasoned that *Nts*^*Cre*^ mice would be useful to examine the molecular expression, projections, activation responses and neurochemistry of this large population of LHA Nts neurons without physiology-disrupting colchicine treatment. First, we verified the fidelity of this model for identifying Nts neurons by colchicine-treating *Nts*^*Cre*^ mice on a Cre-inducible GFP reporter background^35^ (*Nts*^*Cre*^; *GFP* mice). We examined two brain regions that have been shown via Nts-ISH to contain numerous Nts neurons: the subthalamic nucleus (STN) and the LHA^36^. Similarly, *Nts*^*Cre*^; *GFP* mice have dense populations of GFP-labeled neurons within the STN and LHA that co-label with Nts-IF (Fig. 1E–K). Thus, *Nts*^*Cre*^; *GFP* mice reliably identify Nts neurons and can be used to characterize features of LHA Nts neurons.

### *Nts*^*Cre*^; *GFP* Mice Confirm that Some LHA Nts Neurons Project to the Midbrain

**Figure 2:**
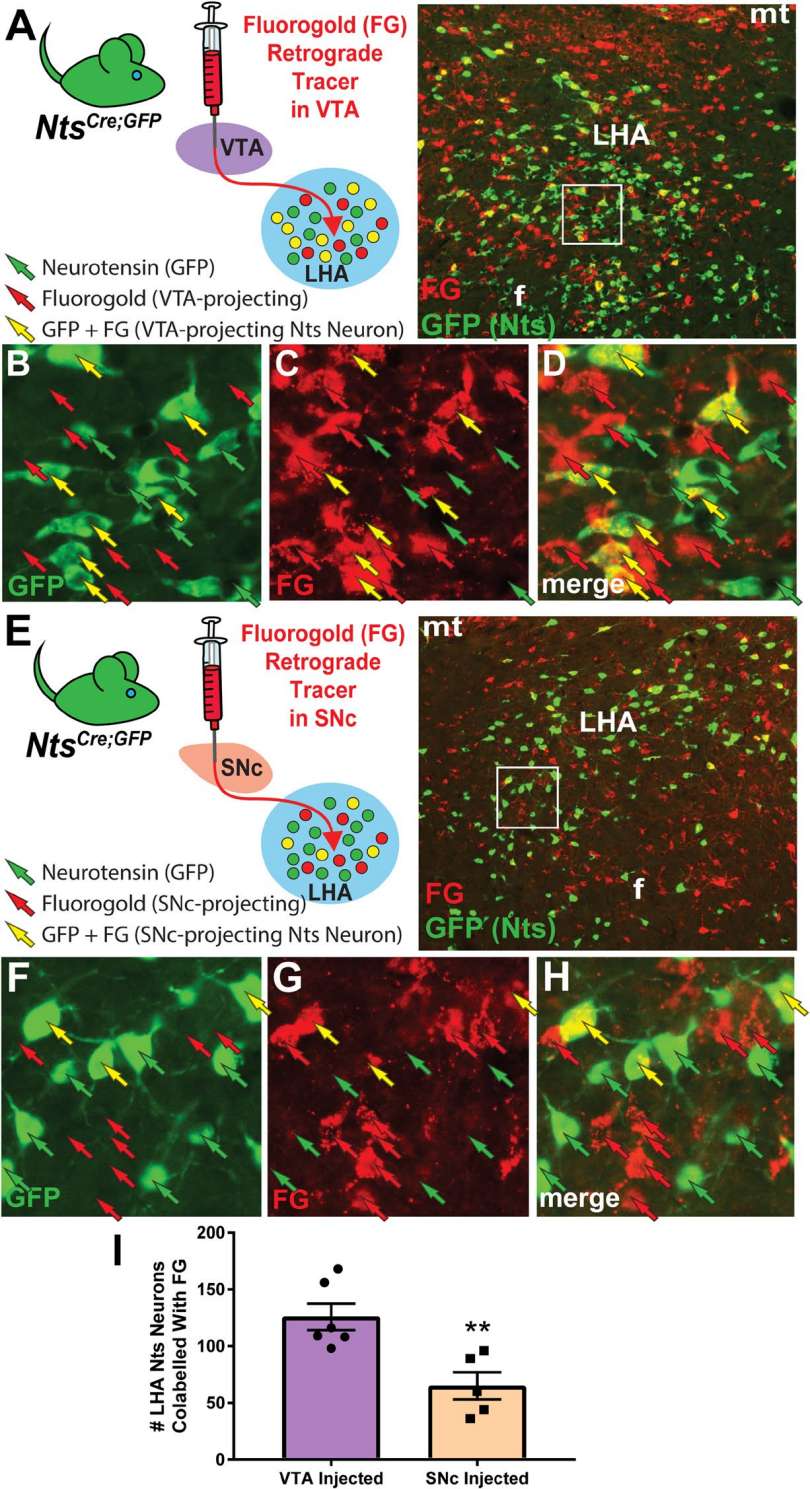
LHA Nts Neurons Project to the VTA and SNc (**A**) *Nts*^*Cre*^; *GFP* mice were injected in the VTA with the retrograde tract tracer FluoroGold (FG). (**B**–**D**) Representative insets from the LHA showing (**B**) GFP-labeled Nts cell bodies (green) and (**C**) cell bodies that have accumulated FG (red) and from the VTA. (**D**) Some GFP-labeled Nts cells contain FG, indicating LHA Nts neurons that project to the VTA (yellow arrows) while GFP-labeled Nts cells lacking FG do not project to the VTA (green arrows). Some non-Nts cells also project to the VTA (red arrows). (**E**) *Nts*^*Cre*^; *GFP* mice received FG into the SNc. F-H) Insets from the LHA show (**F**) GFP-labeled Nts cell bodies (green) and (**G**) cell bodies that have accumulated FG from the SNc (red). (**H**) The LHA contains cells co-labeled with GFP and FG indicating that they project to the SNc (yellow arrows), as well as GFP-labeled Nts neurons that lack FG and do not project to the SNc (green arrows). There are also non-Nts neurons that project to the SNc (red arrows). For (**A**–**H**), VTA-injected n = 12, SNc-injected n = 11. (**I**) Quantitation of the number of LHA Nts cells co-labeled with GFP and FG after FG injection into the VTA (n = 6) or the SNc (n = 5), **p < 0.01 via Student’s t-test. Together, these data demonstrate that some LHA Nts neurons project to the VTA and the SNc, with more projections targeting the VTA. Abbreviations: mt = mammillothalamic tract; f = fornix; LHA = lateral hypothalamic area.

LHA Nts neurons project to two midbrain regions, the ventral tegmental area (VTA) and the substantia nigra compacta (SNc)^26,37^, which regulate motivated behaviors and motor function, respectively^38,39^. We therefore hypothesized that different subsets of LHA Nts neurons might project to the VTA or SNc. First, we identified LHA Nts projecting neurons by injecting *Nts*^*Cre*^; *GFP* mice with FluoroGold (FG) in the VTA (Fig. 2A–D) or the SNc (Fig. 2E–H), which is taken up by terminals and transported retrogradely to the soma of origin. Examination of VTA-injected mice revealed many LHA cell bodies that accumulated FG, some of which also contained GFP and hence identify LHA Nts neurons that project to the VTA (Fig. 2B–D, yellow arrows). Yet, many LHA Nts neurons did not accumulate FG from the VTA (Fig. 2B–D, green arrows). This could be because some LHA Nts neurons do not project to the VTA, or if there was insufficient FG throughout the VTA subregions containing LHA Nts terminals. However, we also found adjacent FG-labeled cells lacking GFP, indicating non-Nts containing LHA cells that provide input to the VTA and that FG coverage was sufficient to label various LHA soma (Fig. 2B–D, red arrows). Analysis of SNc-injected mice revealed similar groups of labeled neurons (Fig. 2F–H). Together, these data verify that that some LHA Nts neurons project to the VTA and SNc, but nearly twice as many project to the VTA vs. the SNc. (Fig. 2I). Our methodology prevented determination of whether any LHA Nts neurons provide collateral projections to these areas. However, the differing number of projections hint that there may be some separate subpopulations of LHA Nts neurons that project to the VTA, SNc, or sites other than the midbrain.

### Nts^LepRb^ Neurons Are a Subset of LHA Nts Neurons that Project to the Midbrain

**Figure 3:**
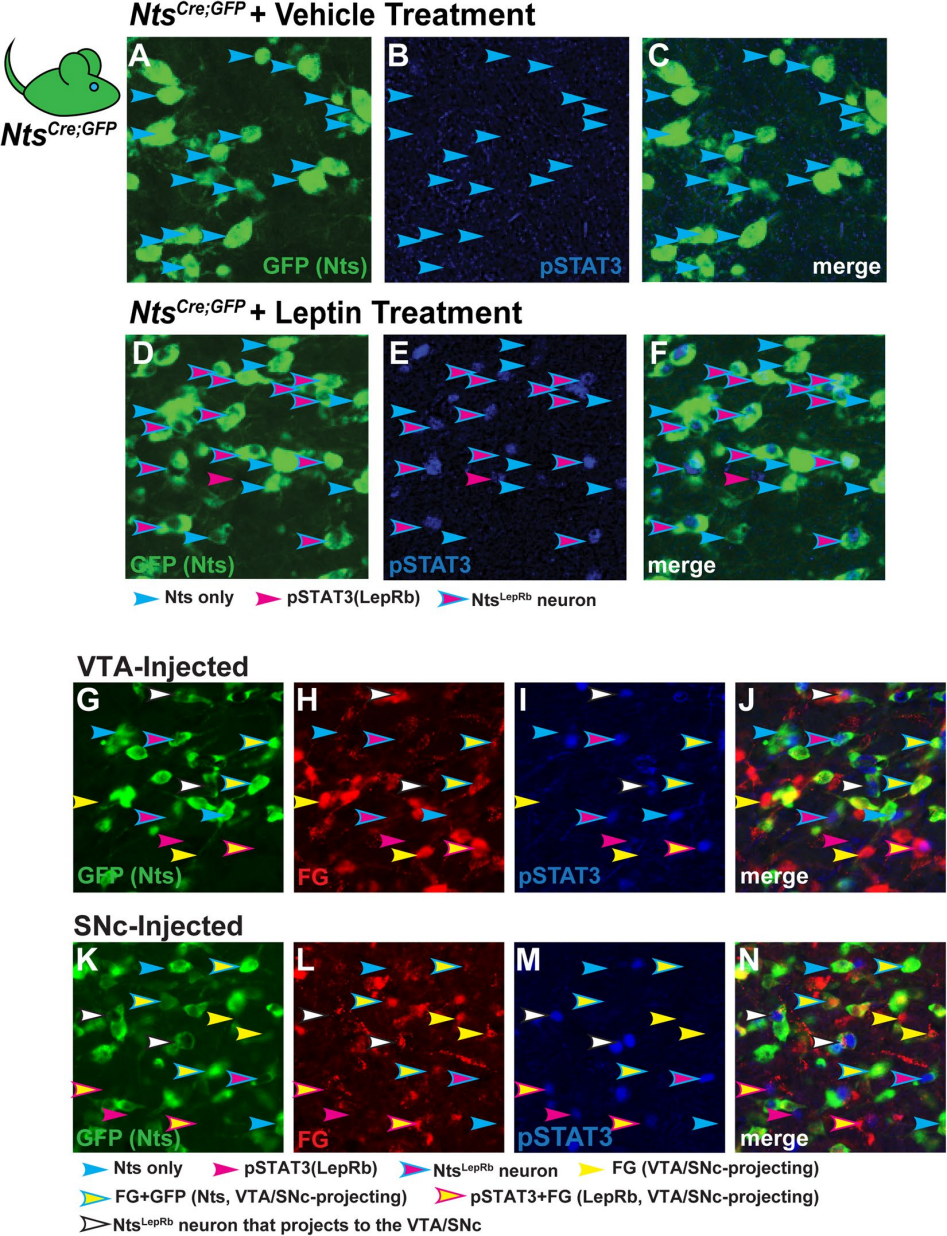
LHA Nts^LepRb^ Neurons Project to the Midbrain *Nts*^*Cre*^; *GFP* mice were treated with (**A**–**C**) vehicle or (**D**–**F**) leptin (5 mg/kg, IP, 2–4 hr) to permit detection of (**A**) GFP-labeled Nts neurons (green) and (**B**) phosphorylated STAT3 (pSTAT3), a marker for leptin-activated LepRb neurons (blue). Cyan arrows label Nts neurons without pSTAT3. Magenta arrows identify pSTAT3 that does not co-label with GFP (e.g. LepRb neurons that do not express Nts). Cyan-outlined magenta arrows identify GFP-labeled Nts neurons that co-localize with pSTAT3 and are Nts^LepRb^ neurons. (**G**–**N**) *Nts*^*Cre*^; *GFP* mice received FG in the VTA or SNc and were treated with vehicle or leptin to permit identification of LepRb neurons via induction of pSTAT3. Examination of the LHA from VTA-injected mice revealed (**G**) GFP-labeled Nts neurons, (**H**) FG-labeled neurons that project to the VTA and (**I**) pSTAT3 neurons. (**J**) Merged panels identify some neurons containing GFP, FG and pSTAT3 that are Nts^LepRb^ neurons that project to the VTA (white arrows). (**K**–**N**) Examination of the LHA from SNc-injected mice reveals (**K**) GFP-labeled Nts neurons, (**L**) FG-labeled neurons that project to the SNc and M) pSTAT3 neurons. (**N**) Merged panels identify some neurons containing GFP, FG and pSTAT3 that are Nts^LepRb^ neurons that project to the SNc (magenta-outlined yellow arrows). Key for other arrows: cyan arrows = Nts-GFP only neurons; magenta arrows = pSTAT3-only (LepRb) neurons; cyan-outlined magenta arrows = Nts^LepRb^ neurons that do not project to the VTA/SNc; yellow arrows = FG-only neurons that project to the VTA/SNc; cyan-outlined yellow arrows = Nts neurons that project to the VTA/SNc but do not contain LepRb; magenta-outlined yellow arrows = VTA/SNc projecting LepRb neurons that do no express Nts; white arrows = VTA/SNc-projecting Nts^LepRb^ neurons. These data demonstrate that at least some Nts^LepRb^ neurons project to the VTA and the SNc. VTA-injected vehicle-treated n = 6; female VTA-injected leptin-treated n = 10; SNc-injected vehicle-treated n = 5; SNc-injected leptin-treated n = 9.

Some LHA Nts neurons can be molecularly distinguished via expression of LepRb (Nts^LepRb^ neurons) and we reasoned that they might project to either the VTA or SNc. To identify Nts^LepRb^ neurons we treated *Nts*^*Cre*^; *GFP* mice with vehicle (Fig. 3A–C) or leptin (5 mg/kg *i.p*. 2–4 hr), which induces phosphorylation of STAT3 (pSTAT3) specifically in LepRb-expressing neurons. Some GFP-labeled LHA Nts neurons contained leptin-induced pSTAT3 and these are Nts^LepRb^ neurons (Fig. 3D–F, cyan-outlined magenta arrows). Yet, many GFP-labeled LHA Nts neurons did not contain pSTAT3 (Fig. 3D–F, cyan arrows). These data confirmed that there are at least two molecularly distinct populations of LHA Nts neurons: one population expresses LepRb and can be revealed by leptin-induced pSTAT3 (the Nts^LepRb^ neurons), but another LHA Nts population lacks LepRb.

Next, we asked whether the molecularly-specified Nts^LepRb^ neurons are also projection-specified. *Nts*^*Cre*^; *GFP* mice were injected with FG to label LHA Nts neurons that project to the VTA or SNc, and also treated with leptin to permit pSTAT3-mediated identification of Nts^LepRb^ neurons. This paradigm identified Nts^LepRb^ neurons that accumulated modest amounts of FG from the VTA (Fig. 3G–J, white arrows) and from the SNc (Fig. 3K–N, white arrows). In each case we also observed Nts^LepRb^ neurons that did not accumulate FG; these may be Nts^LepRb^ neurons that project to the midbrain region that was not injected with FG or to a yet undetermined site outside of the midbrain (Fig. 3G–N, cyan-outlined magenta arrows). As expected, we also observed LHA Nts neurons lacking LepRb, some of which project to the midbrain (Fig. 3G–P, cyan-outlined yellow arrows), but other LHA Nts neurons did not accumulate FG and hence do not project to either midbrain region (Fig. 3G–P, cyan arrows). Together, these data signify that Nts^LepRb^ neurons are a molecularly-specified subset of LHA Nts neurons, some of which project to the VTA and SNc.

### Nts^Dehy^ Neurons Are a Subset of LHA Nts Neurons that Do Not Project to the Midbrain

**Figure 4:**
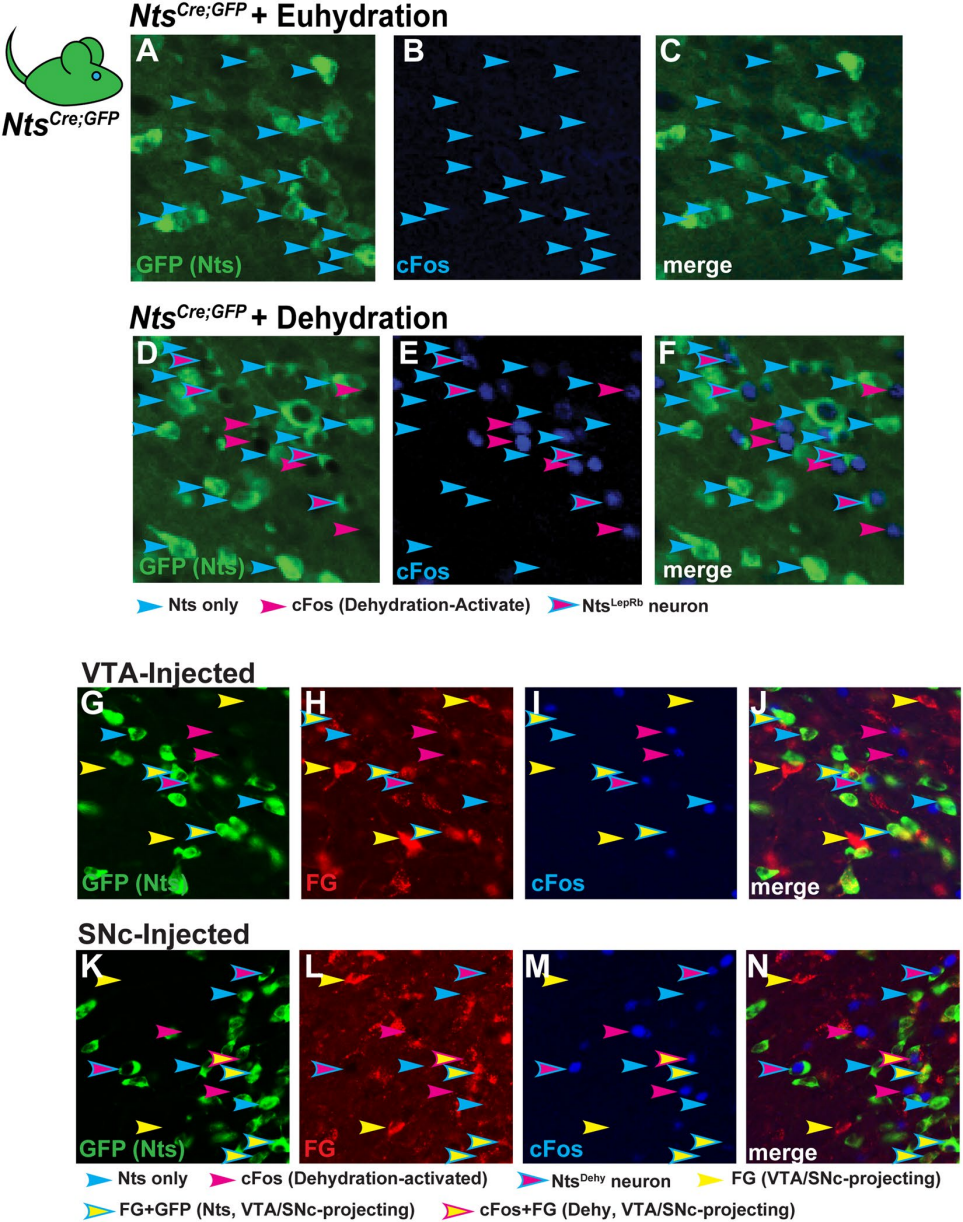
LHA Nts^Dehy^ Neurons Do Not Project to the Midbrain. (**A**–**F**) NtsCre; GFP mice were given *ad libitum* water (Euhydrated) or were dehydrated overnight. Brains were assessed for GFP-labeled Nts neurons (green) and cFos, a marker of recent neuronal depolarization (blue). Cyan arrows label Nts-only neurons and magenta arrows identify dehydration activated neurons that do not express Nts. Cyan-outlined magenta arrows identify Nts-GFP neurons that co-express cFos (Nts^Dehy^ neurons). (**G**–**N**) *Nts*^*Cre*^; *GFP* mice were injected with FG into the VTA or SNc (to identify midbrain projecting neurons) and dehydrated overnight (to identify dehydration-activated neurons via cFos). Assessment of the LHA revealed some Nts^Dehy^ neurons (Cyan-outlined magenta arrows) that did not accumulate FG. Similarly, (**K-N**) Nts^Dehy^ neurons were found within the LHA of SNc-injected mice but none of these contained FG. Key for arrows: cyan arrows = Nts-only neurons; magenta arrows = cFos-only (dehydration-activated) neurons; cyan-outlined magenta arrows = Nts^Dehy^ neurons that do not project to the VTA/SNc; yellow arrows = FG-only neurons that project to the VTA/SNc; cyan-outlined yellow arrows = Nts neurons that project to the VTA/SNc but are not activated by dehydration; magenta-outlined yellow arrows = dehydration-activated VTA/SNc projecting neurons that do no express Nts. No arrows are present to label VTA/SNc-projecting Nts^Dehy^ neurons because no such neurons were found. VTA-injected, euhydrated n = 5; VTA-injected, dehydrated n = 9; SNc-injected, euhydrated n = 5, SNc-injected, dehydrated n = 9.

Since dehydration disrupts bodily osmolality and upregulates Nts mRNA in the LHA^28^, we postulated that it might also modify the activity of LHA Nts neurons. To test this, we provided *Nts*^*Cre*^; *GFP* mice with *ad lib* water (euhydration) or removed water overnight (dehydration), then examined the LHA for GFP and cFos (a marker of recent neuronal depolarization). During euhydration we observed few LHA Nts neurons with cFos, suggesting that LHA Nts neurons are not activated during normal fluid balance (Fig. 4A–C). In contrast, overnight dehydration increased cFos within some (but not all) LHA Nts neurons (Fig. 4D–F, cyan outlined magenta arrows). Thus, dehydration activates a subset of LHA Nts neurons, termed Nts^Dehy^ neurons. We then assessed midbrain projections of Nts^Dehy^ neurons in dehydrated *Nts*^*Cre*^; *GFP* mice previously injected with FG in the VTA or SNc. Although we observed Nts^Dehy^ neurons in the LHA (Fig. 4G–N, cyan-outlined magenta arrows), none accumulated FG from the VTA or SNc. FG accumulation within other LHA neurons confirmed successful retrograde labeling (Fig. 4G–N, yellow and cyan-outlined yellow arrows). Together with Fig. 3, these data indicate that some Nts^LepRb^ neurons project to the midbrain but Nts^Dehy^ neurons do not. Given that Nts^Dehy^ neurons and Nts^LepRb^ neurons have different projection targets, they must comprise distinct subsets of LHA Nts neurons.

### Nts^LepRb^ and Nts^Dehy^ Neurons Are Separate Subpopulations of LHA Nts Neurons

**Figure 5:**
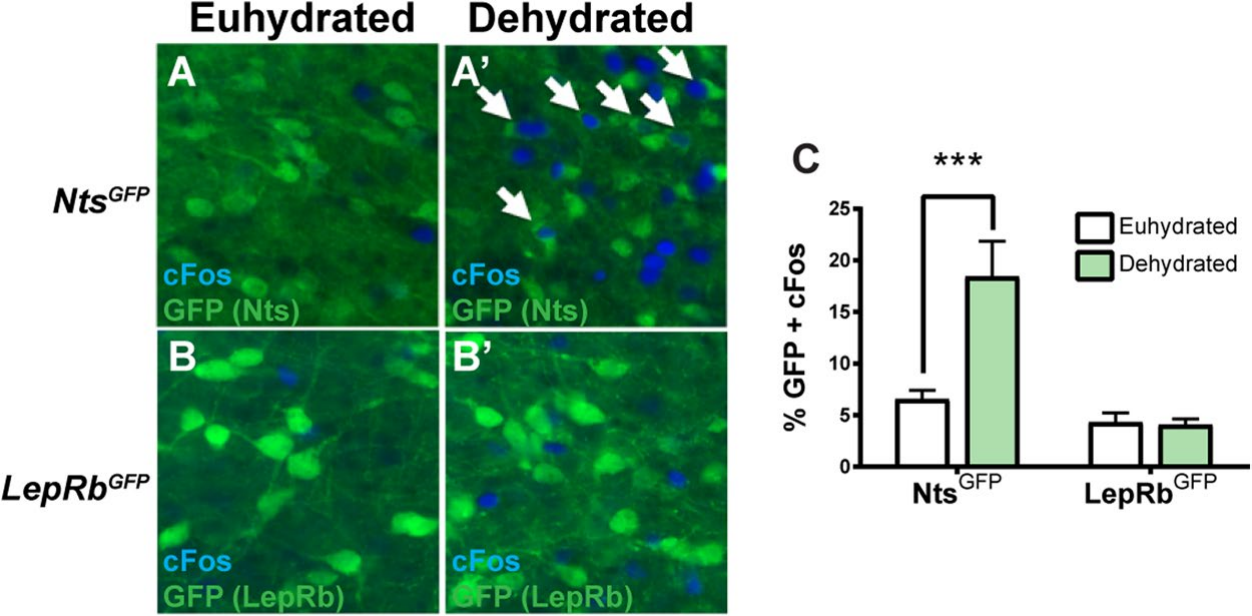
LHA *Nts*^*LepRb*^ Neurons are Distinct from *Nts*^*Dehy*^ Neurons. Mice that express GFP in all LepRb neurons (*LepRb*^*Cre*^; *GFP* mice) and mice expressing GFP in all Nts neurons (*Nts*^*Cre*^; *GFP* mice) were euhydrated or dehydrated overnight. Brains were immunostained for GFP and cFos, a marker of recent neuronal activation. (**A**-**A’**) In *Nts*^*Cre*^; *GFP* mice dehydration induced cFos within LHA Nts neurons. (**B**-**B’**) In *LepRb*^*Cre*^; *GFP* mice dehydration induced cFos in the LHA, but there is no colocalization of cFos with LepRb neurons. (**C**) Percentage of Nts^GFP^ and LepRb^GFP^ in the LHA that contain cFos (e.g. activated neurons) in *Nts*^*Cre*^; *GFP* mice and *LepRb*^*Cre*^; *GFP* mice. Dehydration specifically increases the percentage of GFP + cFos neurons in the LHA, indicating that some LHA^Nts^ neurons are activated by dehydration. Dehydration did not increase the percentage of GFP + cFos in LepRb^GFP^ neurons, which encompass the population of Nts^LepRb^ neurons. These data signify that dehydration does not activate LepRb neurons and therefor the LepRb expressing subpopulation of LHA Nts^LepRb^ neurons is necessarily distinct from LHA Nts^Dehy^ neurons.

We reasoned that if Nts^LepRb^ neurons are distinct from Nts^Dehy^ neurons they would not be activated by dehydration. The requirement for leptin or dehydration to functionally identify Nts^LepRb^ and Nts^Dehy^ neurons meant that we could not simultaneously label these subsets in *Nts*^*Cre*^; *GFP* mice. Instead, to test our hypothesis we assessed cFos in brains from dehydrated *Nts*^*Cre*^; *GFP* mice (where GFP identifies all LHA Nts neurons) and *LepRb*^*Cre*^; *GFP* mice (where GFP identifies all LepRb neurons). Since the majority of LHA LepRb neurons co-express Nts, the *LepRb*^*Cre*^; *GFP* mice identify the Nts^LepRb^ neurons as well as some non-Nts expressing neurons^26^. While dehydration significantly increased the percentage of LHA Nts neurons containing cFos (Fig. 5A,C), it did not alter the proportion of LHA LepRb neurons containing cFos compared to the euhydrated state (Fig. 5B,C). Because LHA LepRb neurons contain the subset of Nts^LepRb^ neurons, the absence of induced LepRb-cFos indicates that Nts^LepRb^ neurons are not activated by dehydration. Conversely, other Nts neurons lacking LepRb can be activated by dehydration. These data indicate that Nts^LepRb^ neurons are functionally distinct from Nts^Dehy^ neurons, as distinguished via their response to dehydration. Furthermore, these subpopulations of LHA Nts neurons are molecularly distinct, such that LepRb expression can be used to distinguish Nts^LepRb^ neurons from Nts^Dehy^ neurons. Taken together, data from Figs 3–5 reveal that LHA Nts neurons are not a homogeneous population but contain molecularly and projection-specified subsets of Nts^LepRb^ neurons and Nts^Dehy^ neurons that are differentially activated in response to changes in energy or fluid balance, respectively.

### Verification of Cre-Dependent Reagents to Determine Classical Neurotransmitter Content

**Figure 6:**
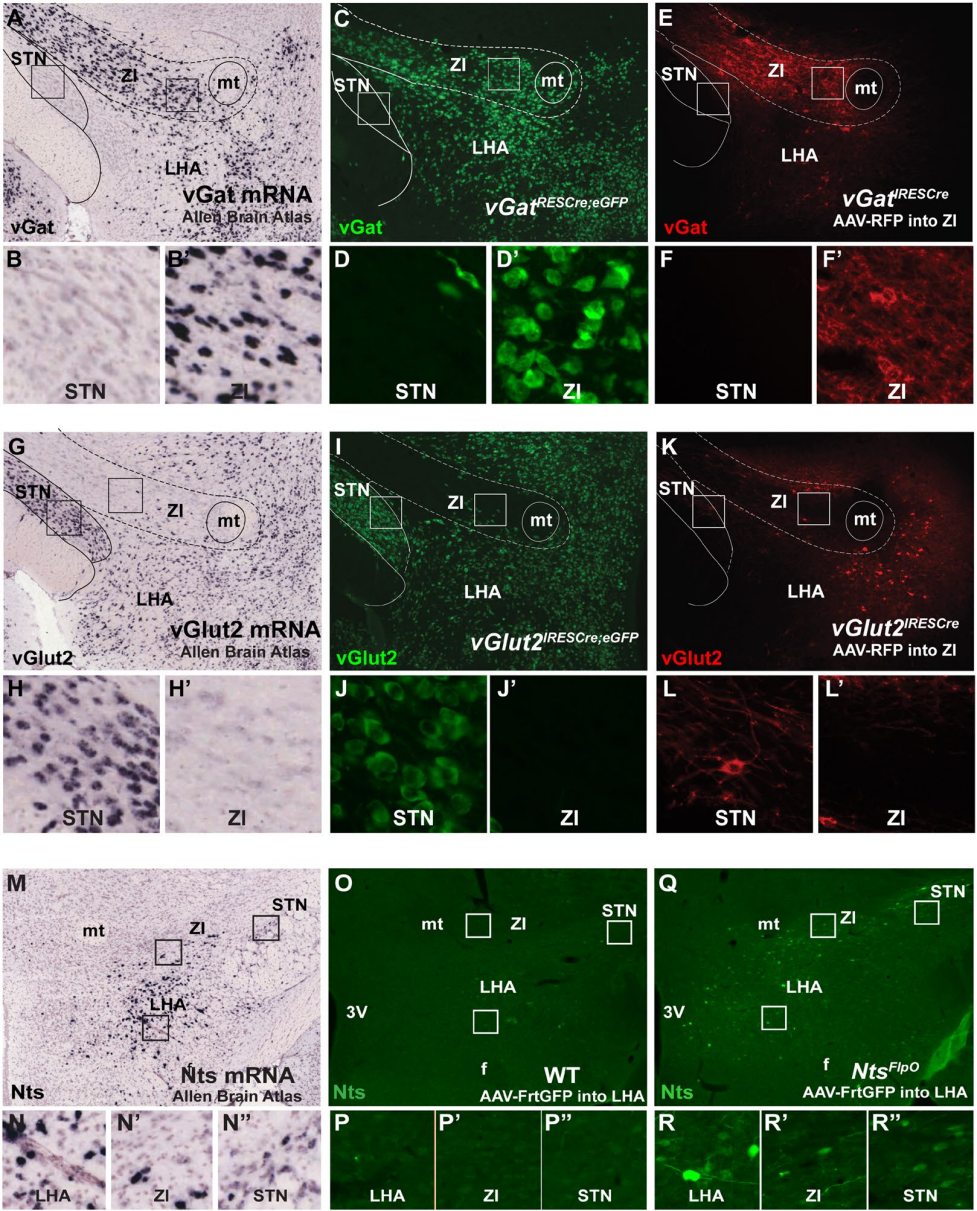
Recombianse Methods to Visualize GABA, Glutamate and Nts Neurons (**A**) ISH for *vGat* from the Allen Brain Atlas^36^. (**B**,**B’**) Digital magnifications of the STN and Zona Incerta from (**A**) show that the STN lacks *vGa*t expression while the ZI contains many *vGa*t-expressing cell bodies. (**C,D**,**D’**) *vGat*^*Cre*^; *GFP* mice show a similar distribution of vGat-GFP labeled cells as *vGat*-ISH (**A**), including few vGat-GFP cells in the STN and many within the ZI. (**E,F**,**F’**) *vGat*^*Cre*^ mice injected with AAV-Lox-RFP have no RFP labeling in the STN but many ZI-labeled RFP cells, and mirror the distribution observed via *vGa*t-ISH and from *vGat*^*Cre*^; *GFP* mice. (**G**) ISH for *vGlut2* from the Allen Brain Atlas. Boxed regions from (**G**) are digitally magnified in (**H**,**H’**) and identify many *vGlut2* cell bodies in the STN but none within the ZI. (**I,J**,**J’**) *vGlut2*^*Cre*^; *GFP* mice identify a similar distribution of GFP-labeled *vGlut2* cells as the *vGlut2*-ISH, including many GFP vGlut2*-*GFP cells in the STN but none in the ZI. (**K,L**,**L’**). Likewise, *vGlut2*^*Cre*^ mice injected in the LHA with AAV-Lox-RFP have some viral spread and vGlut2-RFP labeled cell bodies in the STN but none in the ZI. Collectively these data demonstrate that *vGat*^*Cre*^ and *vGlut2*^*Cre*^ mice can be used with Cre-inducible reporter mice or AAVs to reliably identify vGat and vGlut neurons. (**M–N”**) *Nts*-ISH identifies many Nts-containing cell bodies within the LHA and some within the ZI and STN, courtesy of Allen Brain atlas^36^. (**O–P”**) WT mouse injected with AAV-Frt-GFP in the LHA shows no induced GFP expression within the LHA, ZI or STN. (**Q**–**R”**) *Nts*^*FlpO*^ mouse injected with AAV-Frt-GFP in the LHA shows many GFP-labeled cell bodies around the injection site and within the ZI and STN similar to the distribution of *Nts-*ISH (**M**). These data confirm the specificity of Nts^FlpO^ model and AAV-Frt-GFP to identify Nts neurons in a Cre-independent manner. Abbreviations: mt = mammillothalamic tract; f = fornix; LHA = lateral hypothalamic area, STN = Sub-thalamic Nucleus, ZI = Zona Incerta.

Given the heterogeneity of LHA Nts neurons at the molecular and circuit level, we hypothesized that they might differ in other ways, such as in classical neurotransmitter content. Neuropeptide-expressing neurons may contain GABA or glutamate, release of which determines whether synaptic targets are inhibited or activated, respectively^25^. LHA Nts neurons have been reported as GABAergic^24^ or glutamatergic^40^, thus, we sought to define whether subpopulations might be neurochemically distinguishable. Since GABA and glutamate cell bodies cannot be labeled using immunoreagents, we crossed *vGat*^*Cre*^ and *vGlut2*^*Cre*^ mice with Cre-inducible GFP reporter mice to label these cells with GFP (*vGat*^*Cre*^; *GFP* and *vGlut2*^*Cre*^; *GFP* mice). A limitation of this model is that neurons can alter neurotransmitter expression over lifespan^41^, but recombination during development produces permanent GFP-labeling that may not reflect the neurochemistry of the mature neuron. Thus, we also injected adult *vGat*^*Cre*^ and *vGlut2*^*Cre*^ mice with an AAV Cre-Lox-red fluorescent protein (RFP) so that only actively-expressing GABA and glutamate-expressing neurons undergo recombination to express RFP. Finally, we compared these reporter models with ISH data in brain regions known to primarily contain glutamatergic neurons (the STN) or GABAergic neurons (the zona incerta, ZI) to verify the fidelity of each method for identifying neurotransmitter-expressing neurons in the adult brain. We observed similar distributions of *vgat*-ISH, and GFP- or AAV- Lox-RFP-labeled vGAT neurons in the ZI, but no labeled neurons in the adjacent STN (Fig. 6A–F). In contrast, ISH, GFP- and AAV-Lox-RFP labeling of vGlut2 cells all confirm the absence of glutamatergic neurons in the ZI but each method identified glutamatergic neurons in the STN (Fig. 6G–L). Hence, these genetic and viral methods accurately identify GABA and glutamate neurons and can be used to examine the neurotransmitter content of the LHA.

### *Nts*^*FlpO*^ Mice Identify LHA Nts Neurons Without the Use of Cre

Defining the neurotransmitter content of LHA Nts neurons requires simultaneous labeling of neurons expressing Nts and vGat or vGlut2. Problematically, the use of *vGat*^*Cre*^ and *vGlut2*^*Cre*^ mice necessary for detection of GABA and glutamate neurons precludes simultaneous Cre-dependent detection of Nts using *Nts*^*Cre*^ mice. To overcome this limitation, we generated *Nts*^*FlpO*^ mice to visualize Nts neurons via a non-Cre, FlpO-dependent mechanism. To verify the specificity of the system we injected *Nts*^*FlpO*^ mice and control mice lacking FlpO (WT) with an AAV that drives FlpO-inducible expression of GFP (AAV-Frt-GFP) (Fig. 6M–R”). We observed no GFP-labeled cells in the LHA, ZI and STN when WT mice were injected with AAV-Frt-GFP, despite the many Nts-expressing cells in these regions demonstrated via Nts-ISH (Fig. 6M–P”); these data confirm the FlpO-dependence for GFP expression. By contrast, injection of AAV-Frt-GFP into the LHA of *Nts*^*FlpO*^ mice resulted in GFP-labeled cell bodies within the LHA, ZI and STN (Fig. 6Q,R”). The distribution of GFP cells in *Nts*^*FlpO*^ mice is similar to that of Nts-ISH (Fig. 6M) and *Nts*^*Cre*^; *GFP* mice, although fewer cells are reported; these results are consistent with the limited recombination efficiency of FlpO as compared to Cre^42^.

### Determination of Classical Neurotransmitter Content Within LHA Nts Neurons Using the Dual Recombinase System

**Figure 7:**
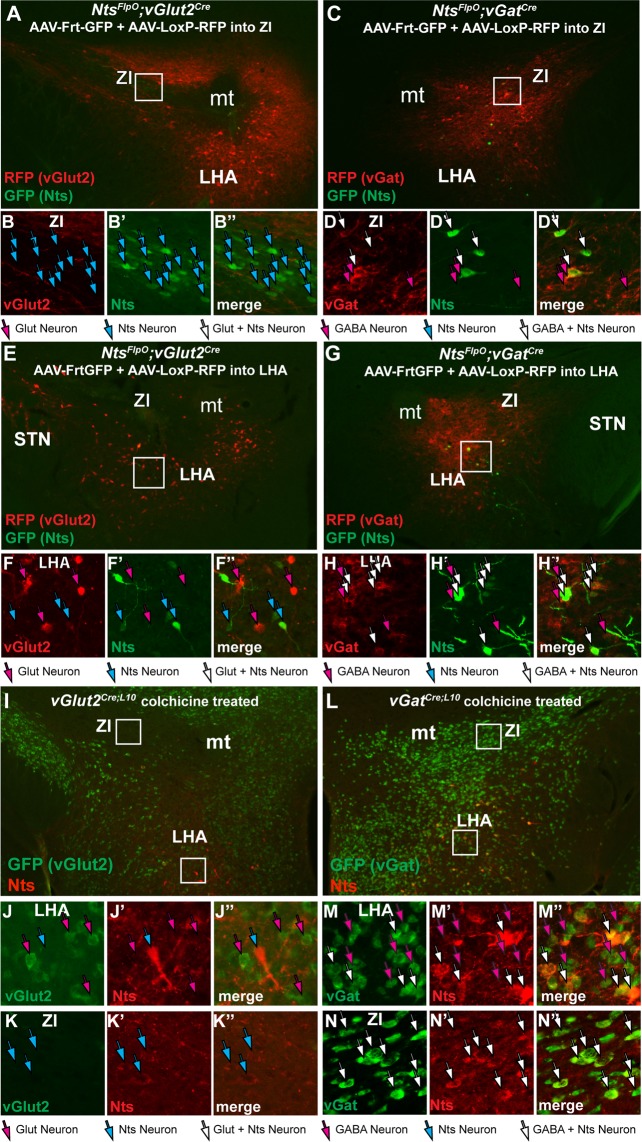
Neurotransmitter Content of LHA Nts Neurons. (**A**,**B”**) *Nts*^*FlpO*^; *vGlut2*^*Cre*^ mice were injected in the ZI with AAV-Frt-GFP (to identify Nts neurons, green) and AAV Cre-RFP (to identify glutamate neurons, red). Digital magnification of the boxed area in A revealed many GFP-Nts neurons (cyan arrows) but no vGlut2 neurons in the ZI, consistent with the GABAergic nature of this brain region. (**C**,**D”**) The same dual AAV injection into the ZI of *Nts*^*FlpO*^; *vGat*^*Cre*^ mice identified vGat/GABA neurons in the ZI (magenta arrows), and all observed GFP-Nts neurons contain vGat2 (white arrows). These data confirm that the dual recombinase method discerns GABA vs glutamate-containing areas of the brain while also permitting identification of Nts neurons. (**E**,**F”**) Dual AAV injection into the LHA of *Nts*^*FlpO*^; *vGlut2*^*Cre*^ mice identifies RFP-vGlut2 neurons (magenta arrows) and GFP-Nts neurons (cyan arrows). No overlapping RFP and GFP neurons were observed, indicating that LHA Nts neurons do not contain glutamate. (**G**,**H”**) Dual AAV injection into the LHA of *Nts*^*FlpO*^; *vGat*^*Cre*^ mice revealed many RFP-vGat neurons (magenta arrows), and also GFP-Nts neurons that overlapped with RFP-vGat cells (white arrows). Together, these data indicate that LHA Nts neurons express GABA but not glutamate. *Nts*^*FlpO*^; *vGlut2*^*cre*^ n = 5, *Nts*^*FlpO*^; *vGat*^*Cre*^ n = 6). (**I**,**N”**) *vGlut2*^*Cre*^; *GFP* mice and *vGat*^*Cre*^; *GFP* mice were treated with colchicine to permit detection of Nts-IF (red) and vGat- or vGlut2-GFP (green). (**J**,**J”**) In *vGlut2*^*Cre*^; *GFP* mice, many GFP-vGlut2 cell bodies are found in the LHA (magenta arrows) along with Nts-IF cell bodies (cyan arrows), but no overlapping cells were found. (**K**–**K”**) The ZI from *vGlut2*^*Cre*^; *GFP* mice contained a few Nts-IF neurons (cyan arrows) but no vGlut2-GFP cells, consistent with the GABAergic neurochemistry of the ZI. (**M**–**M”**) The LHA of *vGat*^*Cre*^; *GFP* mice contained many GFP-vGat cell bodies (magenta arrows) and Nts-IF cells that all co-labeled with GFP-vGat (white arrows). (**N**-**N”**) Similarly, co-labeling was observed in the GABAergic ZI, (**N**-**N”**) but not in *vGlut2*^*Cre*^; *GFP* mice. These data confirm that LHA Nts neurons contain vGat and are GABAergic, but do not contain vGlut/glutamate. *vGlut2*^*Cre*^; *GFP* mice n = 4; *vGat*^*Cre*^; *GFP* mice n = 5. Abbreviations: mt = mammillothalamic tract; f = fornix; LHA = lateral hypothalamic area, STN = Sub-thalamic Nucleus, ZI = Zona Incerta.

We generated dual recombinase mice to simultaneously label Nts cells and vGat or vGlut2-expressing cells by crossing *Nts*^*FlpO*^ mice to *vGat*^*Cre*^ or *vGlut2*^*Cre*^ mice, producing *Nts*^*FlpO*^; *vGat*^*Cre*^ and *Nts*^*FlpO*^; *vGlut*^*Cre*^ mice respectively. To test the fidelity of these dual recombinase mice, we injected them in the ZI with AAV-Frt-GFP (to permit FlpO-mediated expression of GFP that identifies Nts neurons) and AAV-Lox-RFP (to permit Cre-mediated expression of RFP for detection of vGat or vGlut2 neurons). Since the ZI contains primarily GABAergic but not glutamatergic neurons as well as some Nts neurons (Figs 1 and 6), the dual recombinase system should only result in GFP and RFP co-labeling of ZI cells in *Nts*^*FlpO*^; *vGat*^*Cre*^ mice, but not in *Nts*^*FlpO*^; *vGlut*^*Cre*^ mice. As anticipated, dual AAV injection into the ZI of *Nts*^*FlpO*^; *vGlut*^*Cre*^ mice yielded many GFP-labeled Nts neurons, none of which contain RFP-vGlut2 (Fig. 7A,B”, cyan arrows). The GABAergic ZI is in fact devoid of RFP-vGlut2, despite the robust induction of RFP in surrounding regions known to contain glutamate (Fig. 7A). By contrast, in dual AAV-injected *Nts*^*FlpO*^; *vGat*^*Cre*^ mice, we observed that all of the GFP-labeled Nts cells co-label with RFP-vGat, indicating that ZI Nts neurons are GABAergic (Fig. 7C,D”; white arrows indicate co-labeled cells, magenta arrows identify RFP-vGat cells that do not contain GFP-Nts). Taken together, these data confirm that the ZI Nts cells are GABAergic, but not glutamatergic, as would be expected of this primarily GABAergic brain region. Our findings also confirmed the reliability of the dual recombinase system to distinguish classical neurotransmitter content, so we next used it to determine whether subsets of LHA Nts neurons can be discriminated via their classical neurotransmitter expression. Dual AAV injection into the LHA of *Nts*^*FlpO*^; *vGlut*^*Cre*^ mice identified GFP-Nts neurons confined within the perifornical LHA (Fig. 7E,F”, cyan arrows) and RFP-vGlut2 neurons (Fig. 7E,F”, magenta arrows), but we did not observe any LHA cells that co-expressed both labels. By contrast, dual AAV injection into the LHA of *Nts*^*FlpO*^; *vGat*^*Cre*^ mice identified GFP-Nts neurons within the perifornical LHA, most of which co-labeled with RFP-vGat (Fig. 7G,H”, white arrows). We also observed many RFP-vGAT neurons that did not co-label with GFP-Nts (Fig. 7G,H” magenta arrows). Together these data suggest that LHA Nts neurons are predominantly GABAergic, and they comprise a subset within the larger population of LHA GABA neurons.

### Determination of Classical Neurotransmitter Content of LHA Nts Neurons Using Colchicine-Mediated Nts-IF

The dual recombinase method suggests that LHA Nts neurons do not contain glutamate based on the absence of neurons co-expressing both Nts-GFP and RFP-vGlut2. This negative result could also be an artifact, perhaps if there were inefficient AAV-Lox-RFP infection within *Nts*^*FlpO*^; *vGlut*^*Cre*^ mice that resulted in under-detection of LHA glutamate neurons. We therefore sought to validate the classical neurotransmitter content of LHA Nts neurons using an independent strategy that did not depend on AAV-mediated recombination. We attempted to generate dual-reporter mice, but commercially available FlpO reporter lines proved ineffective for simultaneous labeling of Nts and vGat or vGlut2 neurons. This also raised concern that limited efficiency of FlpO-mediated recombination in *Nts*^*FlpO*^ mice might under-report LHA Nts neurons and diminish the likelihood of detecting a small population of glutamatergic LHA Nts neurons.

Alternately, we treated *vGat*^*Cre*^; *GFP* and *vGlut2*^*IRESCre*^; *GFP* mice (validated in Fig. 6) with colchicine, allowing for simultaneous visualization of GFP-labeled vGat and vGlut2 and Nts-IF (as in Fig. 1). Similar to findings using the dual recombinase strategy, we observed numerous Nts-IF cell bodies within the LHA and ZI of colchicine-treated mice (Fig. 7I–N”). While the ZI from *vGlut2*^*Cre*^; *GFP* mice was devoid of GFP-vGlut2 neurons (Fig. 7I,K–K”), *vGat*^*Cre*^; *GFP* mice had numerous GFP-vGat neurons in the ZI, many of which also contained Nts-IF (Fig. 7L,N–N”, white arrows). These data are consistent with the GABAergic phenotype of the ZI and our findings using the dual recombinase system (Fig. 7G,H) that ZI Nts neurons are GABAergic but not glutamatergic. We observed many Nts-IF labeled cell bodies within the LHA, as well as GFP-labeled LHA glutamate neurons (Figure I’) and GFP-labeled GABA neurons (Fig. 7L). Despite the robust GFP-labeling induced in both lines, we did not observe any Nts-IF cell bodies within the LHA that co-localized with GFP-vGlut2 (Fig. 7J–J”). By contrast, essentially all of the LHA Nts-IF cell bodies overlapped with GFP-vGat (Fig. 7M–M”, white arrows), but many GFP-vGat neurons did not contain Nts-IR (magenta arrows). In sum, colchicine-mediated Nts-IR recapitulated our findings using the dual-recombinase system: that LHA Nts neurons are GABAergic but comprise a subset of the larger population of LHA GABA neurons.

## Discussion

**Figure 8:**
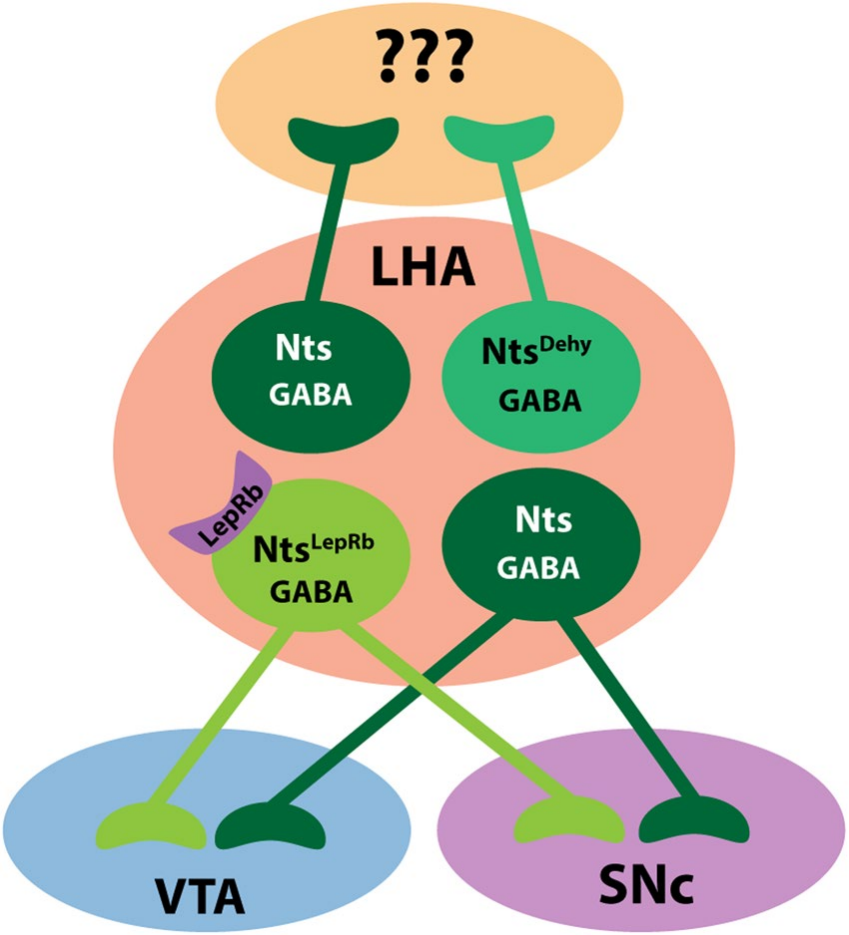
Model of LHA Nts Subpopulations. All LHA Nts neurons co-express the classical neurotransmitter, GABA and some project to the VTA and SNc. Of these, there are distinct subpopulations of LHA Nts neurons that respond to different anorectic cues; some are leptin-sensing (Nts^LepRb^ neurons) and some are dehydration-activated (Nts^Dehy^ neurons). Nts^LepRb^ neurons project the VTA and SNc, where they can access the mesolimbic DA circuit, but Nts^Dehy^ neurons do not. Collectively, these data describe functionally, molecularly and projection-distinct subpopulations of LHA Nts neurons that may contribute to adaptive energy balance.

The LHA is essential for the motivation to eat and drink but the neural mediators of these behaviors have yet to be fully understood. While most LHA populations promote food and liquid intake, LHA Nts neurons divergently regulate ingestive behavior by suppressing feeding and promoting drinking^22,24^. We therefore hypothesized that separate subpopulations of LHA Nts neurons might exist to coordinate opposing feeding and drinking behavior. Here we characterized two separate subpopulations of LHA Nts neurons that are differentially activated by leptin (Nts^LepRb^ neurons) or dehydration (Nts^Dehy^ neurons). While all LHA Nts neurons are GABAergic, the Nts^LepRb^ and Nts^Dehy^ subpopulations differ in molecular expression of LepRb and at the circuit level, and hence can be distinguished via these criteria (Fig. 8). These data demonstrate the heterogeneity of LHA Nts neurons and their specific responsiveness to either energy or fluid balance cues suggest that they may coordinate different ingestive behaviors (feeding vs. drinking).

Here we used *Nts*^*Cre*^ and *Nts*^*FlpO*^ mice to non-invasively and reliably identify Nts neurons, which permitted their study under normal, physiologic conditions. Using *Nts*^*Cre*^ mice, we found that dehydration treatment increased activation from ~6% at baseline to ~18%, demonstrating that ~12% of the LHA Nts neurons are Nts^Dehy^ neurons (Fig. 5C). Additionally, Nts^LepRb^ neurons make up a separate 15% of LHA Nts neurons^22^. While these are modestly sized populations, they can significantly influence homeostasis; for example, mice lacking leptin-regulation via Nts^LepRb^ neurons have impaired response to energy balance cues and diminished dopamine signaling that causes overweight^22,26^. Characterization of the remaining 70% of LHA Nts neurons at the molecular and circuit level may provide insights about their function. For example, some LHA Nts neurons are activated by LPS-mediated inflammation and inhibit local orexin/hypocretin neurons, and these may contribute to illness-behavior^43^. Nts signaling is also implicated in regulation of analgesia, thermoregulation, stress and addiction^44^, so it will be important to determine if/how the remaining LHA Nts neurons contribute to these diverse aspects of physiology. Characterizing the heterogeneity of LHA Nts neurons may also suggest intersectional or pharmacological strategies to target specific subpopulations of LHA Nts neurons, and hence selective physiological outputs.

LHA neurons project widely throughout the brain and differentially modify behavior depending on their targets^44^. Our finding that Nts^LepRb^ neurons, and not Nts^Dehy^ neurons, project to the midbrain suggests that there are distinct LHA Nts neural mechanisms for leptin-mediated suppression of feeding vs. regulation of drinking and fluid balance. Experimental activation of all LHA Nts neurons causes release of Nts to the VTA, and dopamine release into the nucleus accumbens^27^ that can modify motivated intake behavior^27,45,46^. Thus, at least some portion of anorectic leptin regulation via Nts^LepRb^ neurons could occur via direct projections to, and modulation of, mesolimbic dopamine signaling. This is consistent with the requirement of leptin action via Nts^LepRb^ neurons for regulating body weight and the integrity of the mesolimbic dopamine system, which are due in part to Nts signaling via VTA neurons expressing neurotensin receptor-1 (NtsR1)^26,37^. In contrast, Nts^Dehy^ neurons must act via other yet-to-be determined projection targets, and do not directly modulate dopamine signaling to modify physiology. While the function of Nts^Dehy^ neurons and their projection sites remains to be established, the activation of Nts^Dehy^ neurons in response to dehydration suggests that they may coordinate fluid need with the motivation to drink. The discovery of specific subsets of LHA Nts neurons also hints at why experimental activation of all LHA Nts neurons results in diverging ingestive behaviors. Such activation simultaneously induces Nts^LepRb^ neurons that act partially via the VTA (and may be anorectic) as well as the Nts^Dehy^ neurons that regulate separate targets, and it is possible that these populations suppress feeding and promote drinking, respectively^24^. Since Nts^LepRb^ and Nts^Dehy^ neurons are induced by separate physiological cues (leptin or dehydration), it remains to be determined whether there are any physiological situations in which these subpopulations are concurrently activated. In any case, our data confirm that Nts^LepRb^ and Nts^Dehy^ neurons have distinct circuitry, thus projection-specific modulation may be a useful strategy to discern their respective contributions to ingestive behavior.

Despite the molecular and circuit heterogeneity of LHA Nts neurons, they all contain the same classical neurotransmitter, GABA. LHA Nts neurons presumably inhibit synaptic targets via release of GABA, as well as regulating postsynaptic and adjacent neurons via release of Nts. It remains to be determined if GABA and Nts are always co-released from LHA Nts neurons. Indeed, different physiological stimuli bias the release of neurotransmitter vs. neuropeptide signals in some LHA neurons, and the receipt of these messages depends on the repertoire of receptors expressed on target neurons, which can also vary^47–49^. Our finding that LHA Nts neurons are GABAergic is consistent with other reports of overlapping LHA Nts and GABAergic neurons^27,50^ but contrasts with a report of glutamatergic LHA Nts neurons that directly project to the VTA^40^. This discrepancy may be because AAV-Frt-GFP induced GFP expression was confined to the perifornical LHA (~Bregma −1.34 to 1.70^51^), and limited our characterization to this area only. However, the glutamatergic LHA Nts neurons were identified around the “rostral lateral hypothalamus” corresponding to Bregma −0.40, where the rostral LHA merges into the preoptic area, and well beyond the perifornical LHA. Hence, the GABAergic perifornical LHA Nts neurons studied here could be anatomically and neurochemically distinct from LHA Nts neurons of the hypothalamus-preoptic continuum.

While all LHA Nts neurons contain GABA, they are a subset of the vast population of LHA GABA neurons. This may account for the strikingly different behaviors observed after experimental activation of LHA Nts neurons (suppression of feeding, increased drinking) vs. activation of all LHA GABA neurons (increased feeding, drinking and gnawing directed at non-biological objects)^19,20,24,27^. Since activation of LHA Nts and LHA GABA neurons promotes drinking, LHA Nts neurons contribute to at least some of the polydipsic effect. The orexigenic effect observed with activation of all LHA GABA neurons likely masks anorectic effects mediated by the subset of LHA Nts neurons encompassed within them. Our findings agree with reports of functionally-distinct subpopulations of LHA GABA neurons^18^, and LHA Nts neurons are a functionally unique subset within the larger population of all GABA neurons that suppress feeding instead of promoting it. This arrangement may also explain differences in VTA regulation that have been ascribed to these populations. Some LHA GABA neurons project to the VTA, where they disinhibit VTA GABA neurons that in turn releases inhibition of DA neurons to facilitate DA release and feeding^19,52^. Some GABA-containing LHA Nts neurons, including Nts^LepRb^ neurons, also project to the VTA, and their precise synaptic targets are yet to be defined.

Taken together, our data reveal the heterogeneity of LHA Nts neurons. Since LHA Nts neurons are differentially regulated by energy status (leptin) vs. fluid status (dehydration), and comprise separate subpopulations, there may be separate neural mechanisms to coordinate feeding and drinking necessary for survival. If true, then these data may suggest strategies to selectively modify the LHA Nts neurons that control feeding vs. those that modify drinking.

## Materials and Methods

### Animals

Adult male and female mice were used for studies. Some *Nts*^*Cre*^; *GFP* and *LepRb*^*Cre*^; *GFP* mice were generated and treated with euhydration or dehydration at the University of Michigan, under the supervision of the Unit for Laboratory Animal Medicine (ULAM). These procedures were approved by the University of Michigan Institutional Animal Care and Use Committee (IACUC). All other mice were generated from a breeding colony at Michigan State University. MSU mice were cared for by Campus Animal Resources (CAR) and all animal protocols were approved by the Institutional Animal Care and Use Committee (IACUC) at Michigan State University. All mouse experiments were performed in accordance with Association for Assessment and Accreditation of Laboratory Animal Care and National Institutes of Health guidelines. In all cases mice were housed in a 12 h light/12 h dark cycle and had *ad libitum* access to water and chow diet unless otherwise noted.

### Generation of *Nts*^*FlpO*^*Knock*-In Mice

We modified the targeting vector used to generate *Nts*^*Cre*^ mice^26^ to create an *Nts*^*FlpO*^ targeting vector. Briefly, the IRES-Cre was replaced with an IRES-FlpO sequence, such that it is inserted between the stop codon and the polyadenylation site of the sequence encoding the 3’ end of the mouse *Nts* gene. An *frt-*flanked NEO cassette lied upstream of the IRES-FlpO for selection purposes. The linearized *Nts*^*FlpO*^ targeting vector was electroporated into mouse R1 embryonic stem (ES) cells (129sv background) and cells were selected with G418. DNA from ES cell clones was analyzed via qPCR for loss of homozygosity using Taqman primer and probes for the genomic *Nts* insertion sites (Nts-IRES: Forward: TGAAAAGGCAGCTGTATGAAAATAA, Nts-IRES: Reverse: TCAAGAATTAGCTTCTCAGTAGTAGTAGGAA, Nts-IRES: Probe: CCAGAAGGCCCTACATTCTCAAGAGG. *NGF* was used as a copy number control. Putative positive ES clones were expanded, confirmed for homologous recombination by Southern blot and injected into mouse C57BL/6 blastocysts to generate chimeras. Chimeric males were mated with C57BL/6 females (Jackson Laboratory) and germline transmission was determined initially via progeny coat color, then confirmed via conventional PCR for FlpO (as described below).

### Breeding and Genotyping

The *Nts*^*Cre*^; *GFP* and *LepRb*^*Cre*^; *GFP* mice were generated and genotyped as described previously^23^. For all other experiments we utilized *Nts*^*cre*^ mice (Jackson stock #017525) that had been bred onto the C57/Bl6 background (Jackson #000664) mice for at least seven generations. To visualize Nts, vGat and vGlut2-expressing neurons, heterozygous *Nts*^*cre*^ mice, homozygous *Slc32a1*^*tm2(cre)Lowl*^ [Jackson stock #028862] and *Slc17a6*^*tm2(cre)Lowl*^ [Jackson stock # 028863] mice were crossed with homozygous *Rosa26*^*EGFP-L10a*^ mice^35^ and progeny heterozygous for both alleles were studied (*Nts*^*cre*^; *GFP* mice, *vGat*^*Cre*^; *GFP* and *vGlut2*^*Cre*^; *GFP* mice respectively). To simultaneously detect Nts and vGat or vGlut2 we utilized a dual-recombinase strategy. Briefly, we interbred *Nts*^*FlpO*^ mice (to permit FlpO-mediated recombination) and *Slc32a1*^*tm2(cre)Lowl*^ or *Slc17a6*^*tm2(cre)Lowl*^ mice (that enable Cre-mediated recombination) to generate progeny that were heterozygous for FlpO and Cre. These mice were injected with FlpO- and Cre-dependent reporters to visualize Nts and vGAT/vGlut-expressing neurons as described below. Genotyping was performed using standard PCR using the following primer sequences: *Nts*^*cre*^: common forward: 5′ ATA GGC TGC TGA ACC AGG AA, Cre reverse: 5′ CCA AAA GAC GGC AAT ATG GT and WT reverse: 5′ CAA TCA CAA TCA CAG GTC AAG AA. *Rosa26*^*EGFP-L10a*^: mutant forward: 5′ TCT ACA AAT GTG GTA GAT CCA GGC, WT forward: 5′ GAG GGG AGT GTT GCA ATA CC and common reverse: 5′ CAG ATG ACT ACC TAT CCT CCC. Nts^FlpO^: *Nts-FlpO-WT*: Forward: CCAGGAAGATATCCTTGATAACGTCAAT, Reverse: GCAAGAAACATCACATCCAATAAAGCA N, *Nts-FlpO-M:* Forward: TGACCTACCTGTGCTGGATGAT, Reverse: CCACGTTCTTGATGTCGCTGAA. vGat^Cre^: VgatIRESCre Common Forward: CTTCGTCATCGGCGGCATCTG, VgatIRESCre -WT Reverse: CAGGGCGATGTGGAATAGAAA, VgatIRESCre -Mutant Reverse: CCAAAAGACGGCAATATGGT.

### Stereotaxic Injections

Stereotaxic surgeries were performed as described previously^22^ using coordinates from the mouse brain atlas of Paxinos and Franklin^51^. To facilitate detection of Nts containing cell bodies via Nts-IF, adult *Nts*^*cre*^; *GFP*, *vGat*^*Cre*^; *GFP* and *vGlut2*^*Cre*^; *GFP* mice received injections of colchicine (10 μg in a volume of 500 nL) into the lateral ventricle (A/P −0.2, M/L −1.0, D/V −2.1), and were euthanized via cardiac perfusion ~48 hours after treatment. For tract tracing studies, *Nts*^*cre*^; *GFP* mice were injected unilaterally with 75 nL of the retrograde tracer FluoroGold (FG) into the VTA (A/P: −3.2, M/L: +/−0.48, D/V: −4.65) or SNc (A/P: −3, M/L +/−1.3, D/V −4.7), and recovered for 7–10 days to allow for accumulation of FG in cell bodies of origin. Animals were only included in the study if the FG injection was targeted to and confined within the VTA or SNc. To simultaneously identify Nts and vGAT or vGlut, adult *Nts*^*FlpO*^; *vGat*^*Cre*^ and *Nts*^*FlpO*^; *vGlut2*^*Cre*^ mice were injected in the LHA with 400 nL of AAV-Frt-GFP adenovirus (provided by David Olson, University of Michigan) followed by 400 nL of AAV-hM3Dq-mCherry purchased from the UNC Vector Core (here termed AAV-Lox-RFP); these were infused at a rate of 100 nL/minute. LHA coordinates were A/P: −1.34, M/L +/−1.5, D/V −4.9, angle: 6°. Mice recovered for 2–3 weeks to permit sufficient time for recombinase-mediated reporter expression.

### Leptin or Dehydration Treatment

Some FG-injected *Nts*^*cre*^; *GFP* mice were treated with PBS or recombinant mouse leptin (5 mg/kg, i.p.) purchased from the National Hormone and Peptide Program (Los Angeles Biomedical Research Institute, Los Angeles, CA) then were perfused 2–4 hours later to enable detection of leptin-induced pSTAT3. In our hands, both of these leptin treatment times produce comparable pSTAT3. Males and females were studied but no notable differences between sexes were observed so they were pooled for analysis: VTA-injected vehicle-treated n = 6; female VTA-injected leptin-treated n = 10; SNc-injected vehicle-treated n = 5; SNc-injected leptin-treated n = 9.

Other FG-injected *Nts*^*cre*^; *GFP* mice underwent a dehydration-activation paradigm in which they were either had *ad libitum* access to water (euhydration) or the water bottle was removed for 12 hr during the dark cycle (when mice drink most of their daily water). Mice were perfused the following morning and brain sections were stained for cFos, GFP and FG (see below). Due to the lack of observable differences between sexes, males and females were pooled for analysis: VTA-injected, euhydrated n = 5; VTA-injected, dehydrated n = 9; SNc-injected, euhydrated n = 5, SNc-injected, dehydrated n = 9.

Adult male 8–12 wk old *Nts*^*Cre*^; *GFP* and *LepRb*^*Cre*^; *GFP* mice were also treated via euhydration or had water bottles removed for 24 hr (including during the dark cycle) to induce dehydration then were perfused (Euhydrated *Nts*^*Cre*^; *GFP* n = 7, *LepRb*^*Cre*^; *GFP* n = 5; Dehydrated *Nts*^*Cre*^; *GFP* n = 4, *LepRb*^*Cre*^; *GFP* n = 3). Brains were analyzed for cFos and GFP, and 3 representative LHA sections spanning the same Bregma sections from each mouse were analyzed using Photoshop to count the number of GFP-only labeled neurons and GFP neurons containing cFos. Graphed data represent the average percentage of GFP neurons containing cFos out of the total number of GFP-labeled neurons ±SEM. Significant differences between genotypes and treatments were determined via 2-way ANOVA with Bonferroni posttests.

### Immunohistochemistry and Immunofluorescence (IF)

Mice were treated with a lethal dose of *i.p*. pentobarbital and perfused transcardially with either 10% formalin or 4% paraformaldehyde (Sigma-Aldrich 158127) containing 0.4% picric acid (Sigma-Aldrich 197378). Brains were removed, post-fixed in the same fixative overnight at 4 °C, dehydrated with 30% sucrose/PBS for 2–3 days, then sectioned into 30 µm slices using a sliding microtome (Leica). Brain sections were analyzed by immunohistochemistry and/or IF as previously described^22^. For activation studies, brain sections first were exposed to either rabbit-anti pSTAT3 (1:500, Cell Signaling) or goat-anti cFos (1:500, Santa Cruz) followed by incubation with species specific Alexa-488 conjugated (Jackson ImmunoResearch, 1:200) or Alexa-568 conjugated antibodies (LifeTech, 1:200) and visualization with DAB (Sigma). IF labeling was performed exposing sections to primary antibodies, including chicken anti-GFP (1:2000, Abcam), rabbit anti-FG (1:500, Fluorochrome), rabbit-anti Nts (1:500, Phoenix) and/or anti-dsRed (1:1000, Clontech), followed by incubation with species-specific secondary antibodies conjugated to AlexaFluor 488 or 568 fluorophores (1:200, Life Technologies or Jackson ImmunoResearch). Immunolabeled brain sections were analyzed using an Olympus BX53 fluorescence microscope outfitted with transmitted light to analyze DAB-labeling as well as FITC and Texas Red filters for IF. Microscope images were collected using Cell Sens software and a Qi-Click 12 Bit cooled camera, and images were analyzed using Photoshop software (Adobe). We collected images from all mice within an experiment and compared them side-by-side to select the representative images included in the manuscript.

### Quantification of Nts Neuronal LHA –> VTA and LHA –> SNc Projections

FG-injected *Nts*^*cre*^; *GFP* mice were perfused and brains were stained as described above. An intact section between Bregma −1.3 and −1.5 was taken from each sample for quantification. The area medial and dorsal to the Fornix and ventral to the MT was counted to determine the number of FG (VTA/SNc-projecting) and GFP (Nts) labelled neurons in each. Males and females were studied but no notable differences between sexes were observed so they were pooled for analysis: VTA-injected n = 6; female; SNc-injected vehicle-treated n = 5. Graphed data represent the average number of GFP neurons containing FG. Significant differences between brain areas were determined via Student’s t-test and were calculated using GraphPad Prism (GraphPad Software Inc., San Diego, CA). Error bars depict ± standard error of the mean (SEM). **p < 0.01.

### *In Situ* Hybridization (ISH) Data

Some figures include *Nts*, *vGat* and *vGlut2* ISH data from the coronally-sectioned adult mouse brain courtesy of the Allen Brain Atlas (Lein *et al*.^36^), which are identified within our figures as “Courtesy of Allen Brain”. The entire datasets can be referenced via the Allen Brain Atlas website: *Nts*: http://mouse.brain-map.org/experiment/show/73788032; vGat: http://mouse.brain-map.org/experiment/show/72081554; vGlut2: http://mouse.brain-map.org/experiment/show/73818754.

## Data Availability

No datasets were generated during this study. Links to the publicly available datasets of ISH data that were analyzed for this work are listed in the previous section.
